# Supplementary material for: Friendship Bench intervention to address depression and improve HIV care engagement among adolescents living with HIV in Malawi: Study protocol for a pilot randomized controlled trial
Source: PLoS One. 2025 Mar 19;20(3):e0302666. doi: 10.1371/journal.pone.0302666 (PMC11922207; doi:10.1371/journal.pone.0302666)
Supplement: S1 File — (PDF) [file pone.0302666.s002.pdf]

HIV Engagement and Adolescent Depression Support (HEADS-UP): Pilot Trial

Sponsored by:

National Institute of Mental Health (NIMH) at the National Institutes of Health (NIH)

R34 MH130232

Version 1.0
December 15, 2023

HISTORY OF PROTOCOL CHANGES

<u>Version 1.0, 15 Dec 2023</u>

- None

TABLE OF CONTENTS

1.0 ABSTRACT 3

2.0 BACKGROUND, PROBLEM STATEMENT AND JUSTIFICATION 4

2.1 Background and Literature Review 4

2.2 Problem Statement and Justification 5

3.0 OBJECTIVES 5

3.1 Main Objective: 6

3.2 Specific Objectives: 6

4.0 HYPOTHESES 6

5.0 LITERATURE REVIEW 6

6.0 METHODOLOGY 6

6.1 Design of Study: 6

6.2 Place of Study: 7

6.3 Target Population: 7

6.4 Sampling Techniques and Tools 7

6.5 Sample Size Determination 7

6.6 Data Collection Techniques and Tools: 8

6.7 Data Analysis: 8

6.8 Dissemination of Results 9

7.0 ETHICAL CONSIDERATIONS 9

7.1 Human Subjects 9

7.2 Institutional Review Board 9

7.3 Confidentiality, Risks, and Risk Minimization 9

7.4 Benefits to Participants 10

7.5 Costs and Compensation 11

7.6 Informed Consent 11

7.7 Adverse Event Reporting 11

7.8 Study Discontinuation 11

8.0 PERSONAL ROLES AND INSTITUTION 11

9.0 WORK PLAN 12

10.0 BUDGET AND JUSTIFICATION 12

10.1 Budget Justification (USD) 13

11.0 REFERENCES 15

1.0 ABSTRACT

Sub-Saharan Africa is home to the largest population of adolescents living with HIV (ALWH). Engaging ALWH in HIV care is challenging and reflected in lower rates of viral suppression and higher rates of loss to follow-up as compared to adults in the region. Depression has been identified as a significant barrier to initiating and remaining in HIV care. Indeed, this is the case for ALWH in Malawi where estimates of viral suppression range from 40%-78% and estimates of depression range from 18-26%. Resource-appropriate interventions that improve depression and address engagement in HIV care for ALWH are urgently needed.

The Friendship Bench (FB) is an evidence-based depression counseling intervention delivered by trained, supervised lay health workers. It is proven to reduce depression in the general adult population in low-resource settings but has not been adapted to be youth-friendly or enhanced with peer support to facilitate engagement in HIV care among ALWH. FB is based on problem-solving therapy, which offers an ideal framework for youth-friendly adaption and integration of retention peer support into a proven depression treatment model.

During the formative phase of this project, we conducted qualitative research to better understand how depression impacts HIV care engagement amongst ALWH. We used data gathered in the formative phase to adapt the existing, evidence-based FB protocol to meet the developmental needs of ALWH and to enhance the adapted protocol with peer support to facilitate HIV care engagement among ALWH.

In this second phase, we aim to conduct a 3-arm individual-level randomized pilot trial to determine the feasibility, fidelity, and acceptability of the Adapted and Enhanced FB protocols. The second phase is further working towards our long-term goal of adapting, testing, and scaling up resource-appropriate interventions to reduce depression and improve engagement in HIV care amongst ALWH.

2.0 BACKGROUND, PROBLEM STATEMENT, AND JUSTIFICATION

After conducting formative research on depression and HIV care engagement amongst ALWH, we generated evidence for the adaptation and enhancement of the existing, evidence-based FB protocols. This protocol aims to conduct a 3-arm clinic randomized pilot trial to compare the adapted FB and the enhanced FB to standard of care. This pilot trial is the next step towards reaching our long-term goal of adapting, testing, and scaling up resource-appropriate interventions to reduce depression and improve engagement in HIV care amongst ALWH.

2.1 Background and Literature Review

Sub-Saharan Africa (SSA) is home to the largest population of adolescents (10-19 years) living with HIV (ALWH). The 1.46 million ALWH in SSA account for 88% of all ALWH worldwide (1.65 million) and includes individuals who acquired HIV vertically (perinatally) and horizontally (behaviorally).[1] This population is growing due to increasing survival of vertically infected children and increasing rates of horizontal infections among adolescents.[2] In addition, adolescents are the only age group in which AIDS-related deaths are not decreasing in the region.[3] This high burden of morbidity and mortality has led the Joint United Nations Program on HIV and AIDS, the World Health Organization, and NIMH to declare ALWH a priority population requiring immediate attention for research, programming, and policy.[4–8]

Engaging ALWH in HIV care is critical for maximizing the impact of antiretroviral therapy (ART) on health outcomes, life expectancy, and secondary HIV transmission. The challenge of engaging and retaining ALWH in care is reflected in lower rates of viral suppression and higher rates of loss to follow-up for this group compared to adults and children in SSA.[9,10] Indeed, such is the case in Malawi, where 3% of the adolescent population is living with HIV (75,000 of 2,257,834 individuals) and estimates of viral suppression among those on ART range from 40%-78%.[1,11–13]

Poor engagement in care among ALWH has been attributed to the numerous psychosocial and structural challenges that arise from living with a stigmatized, chronic disease in a low-resource setting during a period of developmental transition - challenges that also increase the vulnerability of ALWH to mental health issues such as depression.[14] Estimates of depression for ALWH in Malawi range from 18%-26%, and depression exacerbates poor engagement in care amongst ALWH.[14–19] Consequently, addressing depression among ALWH may not only improve mental health but also ART adherence and viral suppression, affording improved individual health outcomes while also acting as secondary prevention among sexually active adolescents.

Growing literature, mostly involving adults, suggests that mental health interventions delivered by non-specialists in resource limited settings can improve depression outcomes.[20] These task-shifting approaches are critical in countries like Malawi, which has few specialist options for the diagnosis and treatment of common mental disorders.[21] Such task-shifting approaches often incorporate sociocultural frameworks and counseling therapy, moving beyond a purely biomedical approach to mental health.[22–24] However, existing interventions incorporating these approaches have been largely developed for adult populations and there are few data demonstrating their effectiveness among adolescents, particularly ALWH, or if they impact ART adherence.[25–27] Adolescence is a unique period of profound physical, cognitive, and social change where individuals begin to make health-related decisions independently but are still largely influenced by their family members, peers, and intimate partners.[28,29] Navigating these developmental changes is further complicated for ALWH who face issues surrounding inconsistent support from their caregivers, negotiating safe sex, status disclosure to their social ties, stigma related to HIV, and barriers in accessing health services.[30–36] In addition, the experience of ALWH differs based on how they acquired HIV: some adolescents have been managing a chronic disease since birth while others must learn to cope with a more recent infection.[37] Considering these complex needs, there has been a global push for health interventions which are accessible, confidential, and developmentally appropriate, and which provide a safe space where adolescents can share sensitive concerns without judgement.[38] Existing youth-friendly intervention models have largely focused on sexual and reproductive health rather than mental health.[39] Taken together, improving depression outcomes and engagement in care among ALWH requires rigorously adapting and enhancing mental health interventions to address the adolescent population’s unique needs--their mental health *and* HIV care engagement needs.[40]

2.2 Problem Statement and Justification

The Friendship Bench, an evidence-based task-shifting counseling intervention that improves mental health outcomes in low-resource settings, is a superb candidate for such an adaptation. The Friendship Bench intervention was developed over 20 years of community research to provide lay health workers with the tools, training, and supervision to effectively address depression and other common mental health disorders in Zimbabwe.[41] The Friendship Bench was originally intended for use in primary care clinics among the general adult population. Our team has led efforts to pilot test the expansion of the Friendship Bench for adults with HIV, patients with chronic disease, and perinatal women in Malawi[21,42,43] and other researchers are currently adapting the Friendship Bench for HIV negative adolescents in Zimbabwe and Botswana.[44,45] However, the intervention has not been adapted to address the specific needs of ALWH. Our team has also combined the Friendship Bench with ART adherence counseling based on cognitive behavioral therapy and motivational interviewing, using skilled counselors.[46] In Malawi, skilled counselors are not widely available and are often middle aged. Thus, we propose to integrate HIV engagement support into the Friendship Bench intervention using simple, evidence-based peer-delivered strategies.

The Friendship Bench approach is based on problem-solving therapy principles. The Friendship Bench comprises 6 individual sessions delivered by trained, supervised lay counselors attached to a primary care clinic. The Friendship Bench uses problem-solving therapy to improve mental health outcomes by having counselors work with participants to identify a problem and possible solutions.[47,48] The Friendship Bench was tested in a cluster-randomized controlled trial in 24 primary care clinics in Zimbabwe. At 6 months after intervention initiation, participants in clinics randomized to the Friendship Bench had fewer common mental disorders, depressive and anxiety symptoms, and disability severity, compared to those randomized to enhanced usual care.[41]

Peer support is a proven approach for engaging adolescents and young adults in HIV prevention and treatment services. The positive impact of peer support on psychological wellbeing has been well documented among ALWH in SSA and has also been linked to better engagement in HIV care.[14,30,49–53] Utilizing peer supporters is particularly salient for ALWH given the importance of peers for identity creation, acceptance, coping, and normative health behaviors during adolescence.[29,54,55] In addition, previous interventions for ALWH have found that peer supporters are able to reach this population at their point of need in a confidential, safe manner and ensure that they are then linked to relevant services.[27,56–58] Despite these benefits, few studies have mapped support networks of ALWH to understand gaps in sources and types of support, how support needs might vary between vertically and horizontally infected adolescents, what type of peer might be the best to provide support, and what type of training peers might need to meaningfully support emotional well-being, positive health behaviors, and increased HIV care engagement. In addition, there is little information on how peers can engage caregivers in their support activities given that caregiver support is also important for consistent engagement in HIV care for minors.[59–61] Understanding these differences and needs is essential to developing and improving peer support strategies for engagement in care among ALWH.

The formative research generated critical evidence that has allowed us to adapt the FB protocol to meet the developmental and contextual needs of ALWH and enhance the adapted protocol with peer support to facilitate HIV care engagement. The Friendship Bench's problem-solving approach protocol makes it an ideal model to integrate retention in HIV care peer support. Our formative research identified the optimal peer support sessions to be in-person group format. By combining youth-friendly depression treatment with retention peer support, the eventual adapted and enhanced Friendship Bench protocols will address a range of challenges ALWH face during the transition to adulthood and lead to improved mental health and HIV care outcomes.

In this study, we will conduct a 3-arm clinic randomized pilot trial to compare the adapted and the enhanced FB protocols to standard of care. We will determine the feasibility, fidelity, and acceptability of the adapted and enhanced protocols. This pilot trial is the next step towards reaching our long-term goal of adapting, testing, and scaling up resource-appropriate interventions to reduce depression and improve engagement in HIV care amongst ALWH. <u>Information gathered in this proposal will lead to an R01 application for a cluster randomized controlled trial of the adapted and enhanced Friendship Bench interventions to improve depression and engagement in HIV care among ALWH.</u>

3.0 OBJECTIVES

3.1 Main Objective:

- To conduct a pilot trial comparing the Adapted and Enhanced FB protocols to standard of care in addressing depression and HIV care engagement amongst adolescents living with HIV (ALWH)

3.2 Specific Objectives:

- To determine feasibility, fidelity, and acceptability of the Adapted and Enhanced FB protocols
- To compare preliminary effectiveness of the Adapted and Enhanced FB protocols in reducing depression and improving engagement in HIV care

4.0 HYPOTHESES

There are no hypotheses given that the aim of the pilot trial is to assess feasibility, fidelity, acceptability, and preliminary effectiveness of the Adapted and Enhanced FB intervention.

5.0 LITERATURE REVIEW

See section 2.1.

6.0 METHODOLOGY

6.1 Design of Study: We will conduct a three-arm individual-level clinical pilot trial across four clinics. Clinics will be randomized either to adapted Friendship Bench (N = 35 ALWH), enhanced friendship bench (N = 35 ALWH), or standard of care (N = 35 ALWH). Participants will all be recruited and followed for 12 months.

<u>The Adapted Friendship Bench (AFB)</u> will be adapted from the existing Friendship Bench intervention protocol to be youth-friendly and address the unique needs of ALWH.[62-65] Youth friendly adaptation will include selection of young counselors (mixed genders, aged 20-35) who are motivated to work with young people. Counselors will be trained to deliver non-judgmental counseling that respects the privacy and autonomy of ALWH and enables them to make free and informed choices that are relevant to their individual needs. Training will also include discussion of adolescent development and the unique experiences of ALWH related to stigma, disclosure, social relationship issues, and health care access. Counseling sessions will take place in a youth oriented but discrete location within the HIV clinic and be available on weekends to ensure accessibility. AFB will include 6 individual counseling sessions facilitated by counselors attached to one of the study HIV clinics. The first session includes three components called Opening the Mind, Uplifting, and Strengthening, with subsequent sessions building on the first. Opening the Mind refers to the therapeutic process by which, through asking questions, clients are encouraged to open their minds to identify their problems, choose one to work on, identify a feasible solution, and agree on an action plan through an iterative process guided by the counselor. Each structured session lasts 30-45 minutes and will be conducted in a private clinic room in the participant's local language (Chichewa). No specific retention support will be provided, but participants may identify barriers to engagement in HIV care to address during their counseling session. After 4 sessions of individual therapy, the counselor can refer participants not improving or with suicidal ideation to a supervisor trained in mental health to reassess and manage the case. Case management may include additional counseling or pharmacotherapy, at the discretion of the managing clinician.

<u>The Enhanced Friendship Bench (EFB)</u> will include all elements of AFB but will additionally integrate peer support via in person group meetings to facilitate engagement in care for ALWH and depression. Peer supporters will be mature young adults (aged 18-21) who have completed secondary school, are openly living with HIV, and motivated to work with adolescents. The counselors must be willing to maintain participant and colleague confidentiality and sign a confidentiality agreement. Peer Supporters will be trained to deliver youth-friendly group sessions related to HIV care. The content of the 6 peer support sessions includes: Mental Health and HIV, Status Communication, Adherence and Viral Load Testing, Secondary Prevention (Sex and Relationships), Stigma, and Planning for the Future. The 6 sessions will be delivered monthly over 6 months and last 90 minutes each. The group sessions will be in a private space in the clinic in the participant's local language (Chichewa).

Standard of Care (SOC) for mental health in public facilities in Malawi includes options for basic supportive counseling by the primary provider or nurse, medication management by the primary provider, referral to the clinic psychiatric nurse or mental health clinic (most clinics have either an assigned psychiatric nurse or a mental health clinic that visits on a rotating basis), or in more severe cases referral to the psychiatric units at tertiary care hospitals (in the Lilongwe region, this is Bwaila Hospital). For this study, standard care will be enhanced by a trained study nurse who will provide mental health evaluation; brief supportive counseling; information, education, and support on depression; and (if indicated) facilitation of referral to the clinic's psychiatric nurse or mental health clinic or to Bwaila Hospital. The study nurse will have up to 3 follow-up contacts with the participant to assess whether they have followed up on recommended referrals or treatment plans and to assess whether any further outreach is indicated.

6.2 Place of Study: The participating health centers will be the Area 18, Area 25, Kawale, and Lighthouse Health Centers. These sites are all government health facilities located in urban Lilongwe, Malawi with ART clinics on their grounds. We selected clinics well matched in terms of staffing levels, services offered, NGO/ancillary program involvement (e.g., all three sites receive training and supervision support from the Lighthouse Trust NGO in Lilongwe), patient volume, and patient population to minimize any important differences between the sites. UNC-Project Malawi will serve as the administrative home for the study and has longstanding relationships with all 4 proposed study sites. UNC Project-Malawi has previously successfully recruited and enrolled adolescents in several sexual and reproductive health studies as well as HIV-related clinical trials.

6.3 Target Population: ALWH**:** We will recruit 105 ALWH from 4 HIV clinics in Lilongwe, Malawi. We aim to recruit for diversity in age, gender identity, and HIV acquisition type. ALWH will be eligible for the study if they are: (1) age 13-19 ; (2) diagnosed with HIV; (3) scored ≥ 13 on the self-reported BDI-II [19]; (4) willing to provide consent (age 18 or 16-17 years old and married (emancipated minors per Malawi law) or assent with parental consent (age 13-17). All HIV+ individuals will be eligible for recruitment including ART initiators, ART re-initiators and established patients. ALWH screening positive for depression who choose not to participate will be referred to a mental health provider.

Feasibility of ALWH Recruitment: The proposed study will be at conducted at 4 comparable, public health centers in Lilongwe District, Malawi: Area 18, Area 25, Kawale, and Lighthouse. Each of the 4 health centers provides ART services, is located in the urban or peri-urban areas of Lilongwe on a main road, and has a monthly ART clinic volume > 115 individuals (age 10-19) who are either initiating care or established patients.[66] We expect 90% will be between the ages of 13-19. Based on studies among ALHW in Malawi, we conservatively estimate 18% will have depression[19]. Assuming an 80% participation rate, enrolling ~6 ALWH presenting with depression (age 13-19) per site per month (35 ALWH total per site) is feasible in 3 months for a total of 105 ALWH in the entire study. Though current government led data collection efforts for ART care in Malawi include adolescents aged 10-19 and our proposed study includes adolescents in a smaller age window (age 13-19), we believe that our conservative recruitment efforts account this mismatch.

Psychosocial Counselor Recruitment: We will recruit 3 young individuals to be psychosocial counselors at each clinic who will provide the 6 individual counseling sessions to participants (approximately 70 sessions per counselor over 4-6 months). Peer Supporter Recruitment: We will recruit 3 peer supporters for the Enhanced FB arm from the Community Advisory Board, Baylor Teen Club Graduates, and YFHS Clinic Experts. We will aim to select peer supporters who are varied in age, gender, and HIV acquisition status.[67] Supervision for the peer supporters will be provided by the study coordinator.

6.4 Sampling Techniques and Tools

We will recruit ALWH from 4 HIV clinics in Lilongwe, Malawi and each clinic will be randomized to the adapted Friendship Bench, Enhanced Friendship Bench, or standard of care.

6.5 Sample Size Determination

The sample of 105 ALWH (35 per arm) will be sufficient to estimate quantitative measures of feasibility and acceptability with reasonable precision (e.g., confidence intervals around proportions of ±7-9 percentage points across all arms, and ±9-17 percentage points within a given arm).

6.6 Data Collection Techniques and Tools:
To assess feasibility, sources of data include study visit attendance, counseling session attendance, and peer support engagement logs. To assess acceptability, a brief exit interview and behavioral survey will be conducted. To assess fidelity, a counseling session checklist will be collected. Additionally, to assess preliminary effectiveness, we will measure BDI-II and viral load at enrollment, 6-month visit, and 12-month visit. PHQ-9 will also be measured at enrollment, 6-month visit, 12-month visit, and each counseling session. For viral load testing, trained counselors will collect drops of participant blood from a finger stick prick and put the drops on a blood collection card. The blood spot collection card will be sent to a laboratory to assess viral load and then it will be stored in a freezer at UNC Project Malawi until the end of the study. The collection card will not have the participant's name or other identifying information attached, only their study ID number. Blood collection cards will be destroyed at the end of the study.

Record Availability: The site investigator will make all data abstraction documents and records readily available for inspection by the local IRB as requested.

For the brief exit interview, a semi-structured interview guide will be developed to explore acceptability of the FB and peer support sessions. All interviews will be digitally recorded, transcribed in Chichewa, and then translated into English by study staff according to a transcription protocol. All transcripts will then be reviewed by the interviewer for transcription and translation accuracy.

6.7 Data Analysis:

Outcomes**:** The primary goal of this R34 application is to evaluate the feasibility, acceptability, and fidelity of a counseling intervention to improve depression and engagement in HIV care among ALWH. Recognizing the small sample size, indicators of preliminary effectiveness of the intervention for mental health and engagement in care outcomes will be reported as secondary outcomes. Primary and secondary outcomes will be assessed at 6 months and 12 months and compared across the 3 study arms.

Primary Outcomes:

- Feasibility**:** will be defined as the ability to successfully enroll and retain depressed ALWH in the study. Feasibility will be assessed as the number of ALWH enrolled, a comparison of planned to actual enrollment and reasons for non-enrollment, and the proportion of ALWH retained in each arm by the end of the study period. We will also assess the number of sessions that participants attended and peer support engagement during the study period.[68]
- Fidelity: will be defined as adherence to the intervention protocol. Fidelity to content for sessions will be assessed by a member of the research team using a checklist of intervention characteristics. Covering at least 80% of checklist items during each session will be considered fidelity to the intervention protocol.[43, 69]
- Acceptability: will be defined as the ability to deliver and participate in a useful intervention that is appropriate regarding resources and culture. Acceptability will be assessed through brief exit interviews with a sample of ALHW, all study staff, and supervisors. Exit interviews will assess how easy the intervention was to participate in or deliver, the perceived usefulness of the intervention, suggestions for improvement, and will explore contextual factors that impeded or facilitated implementation.[69]

Secondary Outcomes:

- Effectiveness will be assessed through changes in three outcomes: 1) depression symptoms (≥50% change in BDI-II scores from enrollment to the end of the study period), 2) retention in care (whether an ALWH attended at least one appointment per quarter), 3) and viral suppression at 12 months (HIV RNA < 1000 copies/mL).

To assess balance across all 3 study arms at enrollment, we will compare participant characteristics using t-tests for continuous variables and chi-square tests for categorical variables. Quantitative Data: Quantitative measures of feasibility, fidelity, acceptability, and preliminary effectiveness will be summarized using means and standard deviations or proportions and compared across arms using statistical models for continuous or binary outcomes, as appropriate. For feasibility, we will examine the mean number of ALWH enrolled and the proportion of ALWH retained in each of the three study arms (adapted, enhanced, and standard of care). For fidelity, we will examine and compare the proportion of counselors covering at least 80% of checklist items during random direct monitoring sessions across study arms. For acceptability, we will examine and compare the proportion of participants and study staff who found the intervention helpful and easy to deliver across study arms. Changes in depression and engagement in care will be assessed for the 6-month study visit and the 12-month study visit. Generalized estimating equations with an appropriate link for each outcome (i.e., identity link for continuous variables and log link for binary variables) and an exchangeable correlation matrix will be used to examine differences in BDI-II scores, engagement in care, and viral suppression and will account for baseline values of all outcomes and clinic differences. All statistical models will control for any unbalanced confounders. Every effort will be made to minimize missing data, including collecting detailed locator information for the participant as well as for up to two individuals who could help reach the participant should they drop out of contact. However, some missing data is likely to occur. We will examine the data for patterns of missingness and if identified, will conduct sensitivity analyses using inverse probability weights to correct for the bias introduced by the missing data. Qualitative data: Analysis of qualitative exit interview data will involve four steps[70, 71]: 1) reading for content; 2) deductive and inductive coding; 3) data display to identify emerging themes; and 4) interpretation. Codes will be refined during the analysis process and memos will be written for each identified theme.

6.8 Dissemination of Results

We will disseminate our results locally with stakeholders at our clinics in Lilongwe, at UNC-Project Malawi (UNCPM), and through the UNCPM community advisory board. We will also present results at national and international conferences (e.g., IAS, CROI, Academy Health's annual Conference on the Science of Dissemination and Implementation,). Abstracts presented at conferences will also be prepared for publication to disseminate results to the wider scientific community.

7.0 ETHICAL CONSIDERATIONS

7.1 Human Subjects

Safety considerations. Study staff will evaluate patients for safety who endorse any level of suicidal ideation whether or not they enroll in the study. Safety assessment results will be conveyed to the clinical team as appropriate for further follow-up according to the clinic's standard operating procedures.

7.2 Institutional Review Board

Prior to implementation of the pilot trial, all protocol materials will be reviewed by the University of North Carolina Institutional Review Board, in the U.S., and the Malawi National Health Sciences Research Committee (HSRC) before any data collection or analysis occurs. The study will also be reviewed and informed by the existing community advisory board (CAB) of UNC Project in Malawi. The CAB will review the study protocol prior to implementation and provide feedback. The CAB meets monthly. All research procedures will adhere to Malawian and US ethical standards for research involving human subjects.

7.3 Confidentiality, Risks, and Risk Minimization

Risks of loss of confidentiality or social harms to participants will be minimized by 1) training of study staff in the ethical conduct of research; 2) strict protection of confidentiality and personal information; 3) close monitoring of social harms with appropriate IRB reporting.

Discussing personal information – Study participation will include discussion of sensitive topics with ALWH during counseling sessions and peer support sessions. All study counselors will be trained to keep all

information confidential. Research assistants will be trained, and participants informed, that they can pause or discontinue a research interview at any time if they find the topic upsetting.

Data security - All study data, including interview guides, audio recordings of interviews, interview transcribes, and logbooks will be kept in a locked cabinet at the UNC Project data center, where they will be maintained in a locked office at UNC Project accessible only to the principal investigator. Interviews that are transcribed and computerized will be stored on the secure central server at UNC Project. For analysis purposes, data will be de-identified before coding and analysis. Only the principal investigator, data entry personnel, and assigned data analysts will have access to the de-identified electronic study database.

Preventing Discomfort: The surveys, counseling sessions, and peer support activities may cause discomfort for some participants. Efforts will be made to minimize this discomfort by assuring that participants are informed beforehand about the nature of the interaction and that the interaction is completed in a private setting. Participants will be informed that they have the right to decline participation in the study, to refuse to answer any questions, or to withdraw at any time. The consent form will also emphasize that personal information (and inclusion in the study based on HIV and depression status) will be shared during focus groups and confidentially cannot be assured.

Addressing Harms. Our overall goal is to protect the well-being of ALWH, thus if sexual or physical abuse is reported, we will ensure ALWH are referred to a local organization that deals with sexual abuse/violence so that the individual receives appropriate care or to a local social worker. If a study team member comes to believe that a participant is experiencing a crisis (which the participant may or may not recognize) they also may refer to local social workers for counseling or psychosocial support. Examples would include a participant who is having an extreme emotional reaction when discussing the challenges of living with HIV, or a participant whose behavior suggests possible undiagnosed mental illness. These revelations of harm are not common but may occur on a limited basis, necessitating that study protocols be in place to respond appropriately.

At each clinic from which we will recruit participants, we will identify a provider to act as the “clinic lead” for navigation services. This person is a chief point of contact for the study team. When a situation arises where study staff learns of a potential risk of harm to a participant, the following steps will be followed:

(1) The study team member learning of the problem will immediately inform the PI.
(2) If the participant is actively in distress and at the clinic, the study team member will refer the participant to the clinic lead (or, if the lead is not available, to another clinic provider) for immediate evaluation.
(3) If the participant is actively in distress and not at the health center, the study team member will offer to meet the participant and accompanying them to the health facility for immediate referral.
(4) If the participant is not actively in distress, then the study investigators, project manager, and other appropriate project supervisory staff will develop a plan for referring the participant to the clinic lead. As appropriate, the project team may consult the clinic lead in developing the plan. (All consent forms will note that investigators may need to break confidentiality when there is a threat of harm to self or others.) The timing and most appropriate means of effecting a referral will be dependent on the nature of the problem. For example, if a participant reports child abuse in the home, then it would be necessary to involve appropriate social services to investigate the problem. By contrast, if a participant reports that she/he is facing ongoing (but not life threatening) emotional abuse from a partner, then it may make more sense for the navigator to first approach the participant and encourage her/him to meet with a clinic provider or with other social support services in the area.
(5) Study investigators will inform all governing IRBs of the incident and update the IRBs as necessary on the outcomes of efforts to intercede and resolve the harm.

7.4 Benefits to Participants

This study has some minimal risks associated with participation and we anticipate that few participants will experience negative events as a result of taking part in the study. This research will improve our understanding of how an adapted and enhanced FB may improve depression and engagement in HIV care among ALWH. Long-term, supporting ALWH with depression through a counseling intervention carried out by counselors is expected to reduce depression, improve engagement in HIV-care, and ultimately improve adolescent health and HIV outcomes. Therefore, the risk to individual participants in our study is small and the potential benefit to society is substantial.

7.5 Costs and Compensation

Study participants will receive standard compensation in line with UNC Project Malawi practices for their enrollment visit and research outcome interviews.

7.6 Informed Consent

In this study, we are seeking to enroll adolescents 13-19 years to participate. Adolescents who are 18 years or older will provide consent for themselves. Adolescents 13-17 years will provide assent and will have an authorized adult over 18 years provide consent for them prior to participation. The authorized adult can be a parent, a legal guardian, or someone designated as an authorized representative. The research study will be described briefly to all adolescents accessing ART services at Area 18, Area 25, Kawale, or Lighthouse Health Centers in Lilongwe while they wait in the reception area. Once a participant is determined to be eligible for the study a research assistant will complete an informed consent process with each participant in their native language, Chichewa. During the informed consent process, the research assistant will describe the procedures to be followed, the risks and benefits of participation, the duration of participation, and the steps taken to protect participant's confidentiality, particularly with respect to keeping all personal and private information (such as HIV and depression status) private to the extent possible by the study team. The counselor will also emphasize that the consent form says that confidentiality of sensitive information cannot be assured during peer support sessions. Illiterate participants may sign the consent form via thumbprint, in the presence of an impartial witness. Any questions or concerns about privacy will be answered by the study coordinator or referred to the PI, who will address them. To provide protection to this group, we will have rigorous trainings of research staff. We will teach staff to be sure that there is an adequate assessment of understanding. Staff will ensure participants fully understand the content, and time for questions will be encouraged, and answered honestly. We will be clear that refusals will not compromise access to services in any way. We will follow a work practice guideline of when mandatory reporting is required and the best methods to do so, e.g. evidence of exploitation or sexual abuse. Additionally, both sites will adequately map out referrals services to deal with a range of potential needs. All ALWH who screen positive for depression but are not interested in the study will be referred to outpatient psychiatry services. Written informed consent will be obtained from each participant and participants will be provided with a copy of their informed consent forms if they are willing to receive it. Study staff will document the informed consent process.

7.7 Adverse Event Reporting

We do not anticipate any adverse events occurring given the nature of the minimal risks associated with this research. However, in the case that a breach of confidentiality does occur, the study team will immediately inform the IRBs. Specifically:

- Deaths related to study participation shall be reported by the PI to the NIMH PO immediately and no later than within 5 business days of the PI first learning of the death
- SAEs related to study participation shall be reported by the PI to the NIMH PO within 10 business days of the study team becoming aware of the SAE
- Unanticipated Problems Involving Risks to Subjects or Others shall be reported by the PI to the NIMH PO within 10 business days of the study team becoming aware of the problem
- Adverse events and SAEs, including deaths, that are deemed expected and/or unrelated to the study shall be submitted in summary form to the NIMH PO with the annual progress report
- All reported social harms will be documented and communicated to NHSRC and UNC IRB in 7 days
- Protocol violations shall be submitted in summary form to the NIMH PO with the annual progress report
- Suspension or termination of study by IRB shall be reported by the PI to the NIMH program officer (PO) within 3 business days of receipt

7.8 Study Discontinuation

The study may be discontinued at any time by the UNC IRB, the NHSRC, or other government agencies as part of their duties to ensure that research participants are protected.

8.0 PERSONAL ROLES AND INSTITUTION

This proposal is led by Dr. **Bradley Gaynes** (MPI) and Dr. **Nivedita Bhushan** (MPI). Dr. Gaynes is a Professor of Psychiatry and Epidemiology and the Director of Global Mental Health at UNC-CH and has decades of expertise examining the impact of mental health on HIV outcomes, mental health trial design and outcome measurement, and integrating psychotherapy treatment into HIV care in low-resource settings. Dr. Bhushan is a Research Scientist at RTI International and an Early-Stage Investigator with expertise in HIV prevention and treatment among adolescents in SSA as well as the implementation of youth friendly health services. They are supported by Dr. **Brian Pence** (Co-Investigator), a Professor of Epidemiology at UNC-CH who has led research for 15 years on the identification and treatment of mental health disorders among people living with HIV in the US and Africa; Dr. **Kazione Kulisewa** (Co-Investigator), Malawian psychiatrist and long term collaborator with Dr. Gaynes leading mental health reform efforts in the Lilongwe region where this project will be housed; Dr. **Dixon Chibanda** (Consultant), psychiatrist at the University of Zimbabwe, developer of the Friendship Bench and a leader in developing task-shifting models for treating mental health disorders in low-income countries; Dr. **Ruth Verhey** (Consultant), a clinical psychologist, co-director of the Friendship Bench, and an international expert on task-shifting models to effectively treat mental health conditions in low-resource settings; and Dr. **Michael Udedi** (Consultant), the Assistant Director of Clinical Mental Health Services at the Malawi Ministry of Health and an expert on integrating depression treatment into HIV care. These team members have all collaborated previously on mental health research and service initiatives in Malawi.

9.0 WORK PLAN

This pilot trial will be conducted over 18 months. Psychosocial Counselor and Peer Supporter trainings will be conducted in the first 4 months. The pilot trial will be conducted over 12 months, following regulatory approvals.

	Months																	
	**1**	**2**	**3**	**4**	**5**	**6**	**7**	**8**	**9**	**10**	**11**	**12**	**13**	**14**	**15**	**16**	**17**	**18**
Psychosocial Counselor & Peer Supporter Training	■	■																
3-Arm Pilot Implementation and Follow-up			■	■	■	■	■	■	■	■	■	■	■	■				
Pilot Results Analysis and Resulting Manuscript															■	■	■	■

10.0 BUDGET AND JUSTIFICATION

Line Item	Cost per unit (USD)	# and type units	Total Cost	Total Cost (Kwacha)
**1. Study Staff**				
Research Assistant	$450	2 x 12 months	$ 10,800	MK11,870,085
Sub-total			$ 10,800	MK11,870,085
**2. Compensation for study participants and guardians**				
Travel reimbursement for study visits for participants	$10	3 study visits x 105 participants	$ 3,150	MK 3,462,108
Travel reimbursement for enrollment visit for guardians	$10	1 enrollment visit x 105 guardians	$1,050	MK 1,154,036
Travel reimbursement for counseling & peer support sessions	$5	630 sessions	$3,150	MK 3,462,108
Sub-total			$ 7,350	MK 8,078,252
**3. Other Research Costs**				
Translation Fees			$200	MK 219,816
Malawi IRB Fee			$150	MK 164,862

Sub-total			$350	MK 384,678
**Total**			**$18,500**	**MK 20,333,015**
**10% NHSRC fee**			**$1,850**	**MK 2,033,301**

10.1 Budget Justification (USD)

<u>Personnel</u>
Two Research Assistants in Malawi ($10,800 - 100% effort for 12.0 months) – Two research assistants in Malawi will be hired and trained to assist with the pilot trial. $450 salary/month x 12 months x 2 research assistants = $10,800

Total Personnel Costs $ 10,800

<u>Compensation for Study Participants and Guardians</u>
Travel Reimbursement for study visits for participants ($3,150) – A total of 105 participants will be included in our proposed aims. Each participant will receive $10 per study visit (3 per participant) for travel reimbursement.

Travel Reimbursement for enrollment visit for guardians ($1,050) – Each guardian (for 105 adolescent participants aged 13-19) will receive $10 for travel reimbursement for the initial enrollment visit.

Travel Reimbursement for counseling/peer support sessions for participants ($3,150) – Each participant will receive $5 for travel reimbursement for each counseling or peer support session. 35 participants will receive 6 counseling sessions ($1,050) and 35 participants will receive 6 counseling sessions and 6 peer support sessions ($2,100).

Total Compensation for Study Participants and Guardians Costs $ 7,350

<u>OTHER RESEARCH COSTS</u>
Translation and Transcription Fees ($200) – All study materials including informed consent forms, surveys, and interview guides will be translated into Chichewa, the local language in Lilongwe, Malawi. Translation fees $200.

Malawi IRB Fee ($150) – All research in Malawi must receive ethical approval from the National Health Science Research Committee, which charges a $150 fee to review all applications for ethical approval.

Total Other Research Costs $350

TOTAL COSTS: $ 18,500
10% NHSRC Fee: $ 1,850

10.2 Budget Justification (MK)

<u>Personnel</u>
Two Research Assistants in Malawi (MK11,870,085 - 100% effort for 12.0 months) – Two research assistants in Malawi will be hired and trained to assist with the pilot trial. MK 494,108 salary/month x 12 months x 2 research assistants = MK11,870,085

Total Personnel Costs MK11,870,085

<u>Compensation for Study Participants and Guardians</u>
Travel Reimbursement for study visits for participants (MK 3,462,108) – A total of 105 participants will be included in our proposed aims. Each participant will receive MK 10,980 per study visit (3 per participant) for travel reimbursement.

Travel Reimbursement for enrollment visit for guardians ($1,154,036) – Each guardian (for 105 adolescent participants aged 13-19) will receive MK 10,980 for travel reimbursement for the initial enrollment visit.

Travel Reimbursement for counseling/peer support sessions for participants (MK 3,462,108) – Each participant will receive MK 5,490 for travel reimbursement for each counseling or peer support session. 35 participants will receive 6 counseling sessions (MK 1,154,036) and 35 participants will receive 6 counseling sessions and 6 peer support sessions (MK 2,308,072).

Total Compensation for Study Participants and Guardians Costs MK 8,078,252

OTHER RESEARCH COSTS
Translation and Transcription Fees (MK 219,816) – All study materials including informed consent forms, surveys, and interview guides will be translated into Chichewa, the local language in Lilongwe, Malawi. Translation fees MK 219,816

Malawi IRB Fee (MK 164,862) – All research in Malawi must receive ethical approval from the National Health Science Research Committee, which charges a MK 164,862 fee to review all applications for ethical approval.

Total Other Research Costs MK 384,678

TOTAL COSTS: MK 20,333,015
10% NHSRC Fee: MK 2,033,301

11.0 REFERENCES

1. UNICEF. *Key HIV Epidemiology Indicators for Children and Adolescents Aged 0-19, 2000-2018*. (2019).
2. UNAIDS. *Global AIDS Update 2019: Communities at the Centre*. (UNAIDS Geneva, 2019).
3. Slogrove, A. L., Mahy, M., Armstrong, A. & Davies, M.-A. Living and dying to be counted: What we know about the epidemiology of the global adolescent HIV epidemic. *J. Int. AIDS Soc.* **20**, 21520 (2017).
4. Armstrong, A. *et al.* A global research agenda for adolescents living with HIV. *J. Acquir. Immune Defic. Syndr. 1999* **78**, S16 (2018).
5. World Health Organization. *Every Woman, Every Child. Global Strategy for Women's, Children's, and Adolescents' Health (2016-2030): Survive, Thrive, Transform.* (2015).
6. UNAIDS, P. & UNICEF, W. *Start Free, Stay Free, AIDS Free: A Super Fast Track Framework for Ending AIDS in Children, Adolescents and Young Women by 2020*. (UNAIDS, 2016).
7. Wong, V. J., Murray, K. R., Phelps, B. R., Vermund, S. H. & McCarraher, D. R. Adolescents, young people, and the 90–90–90 goals: a call to improve HIV testing and linkage to treatment. *AIDS Lond. Engl.* **31**, S191–S194 (2017).
8. Irvine, C. *et al.* Setting global research priorities in pediatric and adolescent HIV using the Child Health and Nutrition Research Initiative (CHNRI) methodology. *J. Acquir. Immune Defic. Syndr. 1999* **78**, S3 (2018).
9. Hudelson, C. & Cluver, L. Factors associated with adherence to antiretroviral therapy among adolescents living with HIV/AIDS in low-and middle-income countries: a systematic review. *AIDS Care* **27**, 805–816 (2015).
10. Auld, A. F. *et al.* Antiretroviral therapy enrollment characteristics and outcomes among HIV-infected adolescents and young adults compared with older adults—seven African countries, 2004–2013. *MMWR Morb. Mortal. Wkly. Rep.* **63**, 1097 (2014).
11. Umar, E. *et al.* Virological Non-suppression and Its Correlates Among Adolescents and Young People Living with HIV in Southern Malawi. *AIDS Behav.* **23**, 513–522 (2019).
12. Malawi Ministry of Health. *Malawi Population-Based HIV Impact Assessment (MPHIA) 2015-2016: Final Report*. (2018).
13. Nations, U. World population prospects: the 2017 revision, key findings and advance tables. *U. N. N. Y.* (2017).
14. Kim, M. H. *et al.* Factors associated with depression among adolescents living with HIV in Malawi. *BMC Psychiatry* **15**, 264 (2015).
15. Cluver, L. D., Orkin, M., Gardner, F. & Boyes, M. E. Persisting mental health problems among AIDS-orphaned children in South Africa. *J. Child Psychol. Psychiatry* **53**, 363–370 (2012).
16. Domek, G. J. Debunking common barriers to pediatric HIV disclosure. *J. Trop. Pediatr.* **56**, 440–442 (2010).
17. Kamau, J. W., Kuria, W., Mathai, M., Atwoli, L. & Kangethe, R. Psychiatric morbidity among HIV-infected children and adolescents in a resource-poor Kenyan urban community. *AIDS Care* **24**, 836–842 (2012).
18. Kemigisha, E. *et al.* Prevalence of depressive symptoms and associated factors among adolescents living with HIV/AIDS in South Western Uganda. *AIDS Care* 1–7 (2019).
19. Kim, M. H. *et al.* Prevalence of depression and validation of the Beck Depression Inventory-II and the Children's Depression Inventory-Short amongst HIV-positive adolescents in Malawi. *J. Int. AIDS Soc.* **17**, 18965 (2014).
20. Hoeft, T. J., Fortney, J. C., Patel, V. & Unützer, J. Task-sharing approaches to improve mental health care in rural and other low-resource settings: a systematic review. *J. Rural Health* **34**, 48–62 (2018).
21. Udedi, M. *et al.* Integrating depression management into HIV primary care in central Malawi: the implementation of a pilot capacity building program. *BMC Health Serv. Res.* **18**, 593 (2018).
22. Burgess, R. A. Supporting mental health in South African HIV-affected communities: primary health care professionals' understandings and responses. *Health Policy Plan.* **30**, 917–927 (2015).
23. Petersen, I. *et al.* A task shifting approach to primary mental health care for adults in South Africa: human resource requirements and costs for rural settings. *Health Policy Plan.* **27**, 42–51 (2012).
24. Petersen, I., Hancock, J. H., Bhana, A. & Govender, K. Closing the treatment gap for depression co-morbid with HIV in South Africa: Voices of afflicted women. (2013).
25. Ridgeway, K. *et al.* Interventions to improve antiretroviral therapy adherence among adolescents in low- and middle-income countries: A systematic review of the literature. *PLoS ONE* **13**, (2018).
26. Govindasamy, D. *et al.* Uptake and yield of HIV testing and counselling among children and adolescents in sub-Saharan Africa: a systematic review. *J. Int. AIDS Soc.* **18**, 20182 (2015).

27. MacPherson, P. *et al.* Service delivery interventions to improve adolescents' linkage, retention and adherence to antiretroviral therapy and HIV care. *Trop. Med. Int. Health TM IH* **20**, 1015–1032 (2015).
28. Blakemore, S.-J. & Mills, K. L. Is adolescence a sensitive period for sociocultural processing? *Annu. Rev. Psychol.* **65**, 187–207 (2014).
29. Umberson, D., Crosnoe, R. & Reczek, C. Social Relationships and Health Behavior Across the Life Course. *Annu. Rev. Sociol.* **36**, 139–157 (2010).
30. Denison, J. A. *et al.* "The sky is the limit": adhering to antiretroviral therapy and HIV self-management from the perspectives of adolescents living with HIV and their adult caregivers. *J. Int. AIDS Soc.* **18**, (2015).
31. Pantelic, M., Boyes, M., Cluver, L. & Meinck, F. HIV, violence, blame and shame: pathways of risk to internalized HIV stigma among South African adolescents living with HIV. *J. Int. AIDS Soc.* **20**, 21771 (2017).
32. Cluver, L. D., Gardner, F. & Operario, D. Effects of stigma on the mental health of adolescents orphaned by AIDS. *J. Adolesc. Health* **42**, 410–417 (2008).
33. Toska, E., Cluver, L. D., Hodes, R. & Kidia, K. K. Sex and secrecy: How HIV-status disclosure affects safe sex among HIV-positive adolescents. *AIDS Care* **27**, 47–58 (2015).
34. Hazra, R., Siberry, G. K. & Mofenson, L. M. Growing Up with HIV: Children, Adolescents, and Young Adults with Perinatally Acquired HIV Infection. *Annu. Rev. Med.* **61**, 169–185 (2010).
35. Casale, M. The importance of family and community support for the health of HIV-affected populations in Southern Africa: what do we know and where to from here? *Br. J. Health Psychol.* **20**, 21–35 (2015).
36. Casale, M. *et al.* Direct and indirect effects of caregiver social support on adolescent psychological outcomes in two South African AIDS-affected communities. *Am. J. Community Psychol.* **55**, 336–346 (2015).
37. Sherr, L., Cluver, L. D., Toska, E. & He, E. Differing psychological vulnerabilities among behaviourally and perinatally HIV infected adolescents in South Africa - implications for targeted health service provision. *AIDS Care* **30**, 92–101 (2018).
38. World Health Organization. *Making health services adolescent friendly: developing national quality standards for adolescent friendly health services*. (2012).
39. Hawke, L. D. *et al.* What makes mental health and substance use services youth friendly? A scoping review of literature. *BMC Health Serv. Res.* **19**, 257 (2019).
40. Patel, V., Flisher, A. J., Hetrick, S. & McGorry, P. Mental health of young people: a global public-health challenge. *The Lancet* **369**, 1302–1313 (2007).
41. Chibanda, D. *et al.* Effect of a primary care–based psychological intervention on symptoms of common mental disorders in Zimbabwe: a randomized clinical trial. *Jama* **316**, 2618–2626 (2016).
42. LeMasters, K. *et al.* 'Pain in my heart': Understanding perinatal depression among women living with HIV in Malawi. *PLOS ONE* **15**, e0227935 (2020).
43. Gaynes, B. N. *et al.* The Sub-Saharan Africa Regional Partnership (SHARP) for Mental Health Capacity-Building Scale-Up Trial: Study Design and Protocol. *Psychiatr. Serv. Wash. DC* **72**, 812–821 (2021).
44. Cowan, F. M. *et al.* The Friendship Bench for Adolescents: Evaluating Strategies for Scaling Interventions to Treat Common Mental Disorders among Adolescents in Zimbabwe. *UK Research and Innovation Gateway* https://gtr.ukri.org/projects?ref=MR%2FP012485%2F1.
45. Brooks, M. *et al. P11 Adapting a lay counselor mental health intervention for adolescents in botswana*. (BMJ Specialist Journals, 2019).
46. Abas, M. *et al.* Feasibility and Acceptability of a Task-Shifted Intervention to Enhance Adherence to HIV Medication and Improve Depression in People Living with HIV in Zimbabwe, a Low Income Country in Sub-Saharan Africa. *AIDS Behav.* **22**, 86–101 (2018).
47. Bell, A. C. & D'Zurilla, T. J. Problem-solving therapy for depression: a meta-analysis. *Clin. Psychol. Rev.* **29**, 348–353 (2009).
48. Nezu, A. M. & Perri, M. G. Social problem-solving therapy for unipolar depression: an initial dismantling investigation. *J. Consult. Clin. Psychol.* **57**, 408 (1989).
49. Mills, E. J. *et al.* Interventions to promote adherence to antiretroviral therapy in Africa: A network meta-analysis. *Lancet HIV* **1**, e104–e111 (2014).
50. West, N. *et al.* Mental health in South African adolescents living with HIV. *AIDS Care* (2018).
51. Kim, M. H. *et al.* High self-reported non-adherence to antiretroviral therapy amongst adolescents living with HIV in Malawi: barriers and associated factors. *J. Int. AIDS Soc.* **20**, 21437 (2017).

52. Osok, J., Kigamwa, P., Stoep, A. V., Huang, K.-Y. & Kumar, M. Depression and its psychosocial risk factors in pregnant Kenyan adolescents: a cross-sectional study in a community health Centre of Nairobi. *BMC Psychiatry* **18**, (2018).
53. Snyder, K. *et al.* Preliminary results from Hlanganani (Coming Together): A structured support group for HIV-infected adolescents piloted in Cape Town, South Africa. *Child. Youth Serv. Rev.* **45**, 114–121 (2014).
54. Petersen, I. *et al.* Psychosocial challenges and protective influences for socio-emotional coping of HIV+ adolescents in South Africa: a qualitative investigation. *AIDS Care* **22**, 970–978 (2010).
55. Kyngäs, H. Support network of adolescents with chronic disease: Adolescents' perspective. *Nurs. Health Sci.* **6**, 287–293 (2004).
56. van Wyk HonsBSc, M. & Stephanie Luca HonsBSc, M. A. Systematic review of peer support interventions for adolescents with chronic illness. *Int. J. Child Adolesc. Health* **7**, 183 (2014).
57. Willis, N. *et al.* Effectiveness of community adolescent treatment supporters (CATS) interventions in improving linkage and retention in care, adherence to ART and psychosocial well-being: a randomised trial among adolescents living with HIV in rural Zimbabwe. *BMC Public Health* **19**, 117 (2019).
58. Mark, D. *et al.* Peer Support for Adolescents and Young People Living with HIV in sub-Saharan Africa: Emerging Insights and a Methodological Agenda. *Curr. HIV/AIDS Rep.* **16**, 467–474 (2019).
59. Bhana, A. *et al.* The VUKA family program: piloting a family-based psychosocial intervention to promote health and mental health among HIV infected early adolescents in South Africa. *AIDS Care* **26**, 1–11 (2014).
60. Cluver, L. D. *et al.* Achieving equity in HIV-treatment outcomes: can social protection improve adolescent ART-adherence in South Africa? *AIDS Care* **28**, 73–82 (2016).
61. Winskell, K., Miller, K. S., Allen, K. A. & Obong'o, C. O. Guiding and supporting adolescents living with HIV in sub-Saharan Africa: The development of a curriculum for family and community members. *Child. Youth Serv. Rev.* **61**, 253–260 (2016).
62. Tylee, A., Haller, D. M., Graham, T., Churchill, R. & Sanci, L. A. Youth-friendly primary-care services: how are we doing and what more needs to be done? *Lancet Lond. Engl.* **369**, 1565–1573 (2007).
63. Barden-O'Fallon, J., Evans, S., Thakwalakwa, C., Alfonso, W. & Jackson, A. Evaluation of mainstreaming youth-friendly health in private clinics in Malawi. *BMC Health Serv. Res.* **20**, 79 (2020).
64. Dahourou, D. L. *et al.* Transition from paediatric to adult care of adolescents living with HIV in sub-Saharan Africa: challenges, youth-friendly models, and outcomes. *J. Int. AIDS Soc.* **20**, 21528 (2017).
65. Zanoni, B. C., Sibaya, T., Cairns, C. & Haberer, J. E. Barriers to Retention in Care are Overcome by Adolescent-friendly Services for Adolescents Living with HIV in South Africa: A Qualitative Analysis. *AIDS Behav.* **23**, 957–965 (2019).
66. Government of Malawi Ministry of Health. *Integrated HIV Program Report 2019*. (2019).
67. McBride, K. *et al.* ART Adherence Among Malawian Youth Enrolled in Teen Clubs: A Retrospective Chart Review. *AIDS Behav.* **23**, 2629–2633 (2019).
68. Pence, B. W. *et al.* Integrating depression management into HIV care in Lilongwe, Malawi: Feasibility and impact. (2021).
69. Stockton, M. A. *et al.* A Mixed-Methods Process Evaluation: Integrating Depression Treatment Into HIV Care in Malawi. *Glob. Health Sci. Pract.* (2021).
70. Saldaña, J. *The coding manual for qualitative researchers*. (Sage, 2015).
71. Gibbs, G. R. Thematic coding and categorizing. *Anal. Qual. Data Lond. Sage* 38–56 (2007).
