# Supplementary material for: Friendship Bench intervention to address depression and improve HIV care engagement among adolescents living with HIV in Malawi: Study protocol for a pilot randomized controlled trial
Source: PLoS One. 2025 Mar 19;20(3):e0302666. doi: 10.1371/journal.pone.0302666 (PMC11922207; doi:10.1371/journal.pone.0302666)
Supplement: Attachment — Submitted filename: Response to Reviewers_19Aug2024.docx [file pone.0302666.s004.docx]

August 19, 2024

Dear Reviewers,

We are pleased to resubmit our manuscript, “Friendship Bench Intervention to Address Depression and Improve HIV Care Engagement Among Adolescents Living with HIV in Malawi: Study Protocol for a Pilot Randomized Controlled Trial”. We greatly appreciate your thoughtful reviews and addressing your comments has strengthened our manuscript. We have responded to the comments as requested and made all changes to the manuscript in tracked changes. Our detailed responses to your comments can be found in the table below.

Sincerely,

Authors

**Reviewer 1**

| **Comment** | **Response** |
| --- | --- |
| 1. FB is briefly mentioned in the introduction, but it’s not clear what the intervention is. A sentence or two after the first mention is necessary. | We thank the reviewer for this comment. We added one sentence after the first mention of FB in introduction:  **The original Friendship Bench consists of six individual counseling sessions plus optional group peer support.** |
| 2. Page 5, line 6: “In the prior phase of the study,” – There has been no mentioned of a “prior phase of the study”. Any earlier piloting of the intervention/study by the researchers should be mentioned in the methodology section. It needs to be made clear what formative work has been done/you plan to do to adapt the FB intervention to the local setting. | We thank the reviewer for raising this point. We used the ADAPT-ITT framework and this pilot RCT serves as step 7-8 in the framework. We added more information in text:  **In the prior phase of the study, we conducted formative research (in-depth interviews, focus groups, and social support mapping sessions) with ALWH, health care providers, caregivers, and previous young participants and implementors of a FB intervention for perinatal women living with HIV and depression, to examine ALWHs’ experiences of depression, its impact on HIV care engagement, and intervention preferences. Drawing from formative work, we adapted the original Friendship Bench intervention protocol for ALWH to meet their developmental and contextual needs and further enhanced the intervention with peer support to facilitate engagement in HIV care [37,38]. In this study protocol, we describe a pilot RCT phase where we evaluate the adapted and enhanced FB.** |
| 3. The following sentence is confusing and a little too long.  While pharmacological treatments are challenging due to cost, availability of designated professionals 14 and mainly reserved for cases of clinically diagnosed, moderate to severe depression, psychological 15 interventions (known as psychotherapy) are preferable and widely implemented for adolescents and 16 young adults with or without HIV in low- and middle-income countries (Bhana et al., 2020, 2021; 17 Sequeira et al., 2022). | We thank the reviewer for this comment. We edited the sentence as below:  **Pharmacological treatments are challenging due to cost and availability of designated professionals and are often reserved for treating moderate to severe cases of depression. In contrast, psychological interventions are preferable and widely implemented for adolescents and young adults with or without HIV in resource-limited countries [10–12].** |
| 4. There seems to be a word missing after “not been”. Page 4, line 4: It is currently being adapted for adolescents but has not been specifically for ALWH in Botswana (Brooks et al., 2019). | We thank the reviewer for this comment. We edited the sentence as below:  **It is currently being adapted for adolescents in Botswana but has not been specifically adapted for ALWH.** |
| Considerations: The one major point that is lacking from the protocol is considerations around scaling the intervention. The one barrier that comes to mind is the availability of healthcare workers to provide the intervention outside a research context. Also, will the peer component be funded, in which case, is there scope for this to be a paid mandate in Malawi. How has the model scaled among adults in other countries? | We thank the reviewer for raising this important point. The availability of lay workers and peer supporters to implement the intervention outside of a research context is indeed critical for future scale-up. Our current pilot work is focused on evaluating the feasibility, acceptability, fidelity, and preliminary effectiveness of implementing this evidence-based intervention for ALWH in Malawi. We plan to explore facilitators and barriers to scale-up during out exit interviews with study staff, clinicians, and Malawi Ministry of Health officials. |

**Reviewer 2**

| **Comment** | **Response** |
| --- | --- |
| 1. Page 3: line 7-9 Indeed, comorbid depression is prevalent and burdensome amongst ALWH in SSA and depression is a significant threat to engagement and retention in HIV care as well as worsening HIV related outcomes among those receiving HIV treatment” Comment: Are there any stats from past or recent studies showing the burden of depression among adolescents and more specifically among ALWH or HIV care disengagement rates among ALWH with depression or mental health challenges? | We thank the reviewer for this comment. We added some descriptive statistics for the prevalence of depressive symptoms among ALWH in SSA.  **Indeed, comorbid depression is prevalent and burdensome amongst ALWH in SSA with estimates of depressive symptoms among ALWH ranging from 12% to 45% [4].** |
| 2. Page 4: line 4 It is currently being adapted for adolescents but has not been specifically for ALWH in Botswana. Comment: The sentence is unclear, reference to Botswana is a bit confusing. Please rephrase. | We thank the reviewer for this comment. We edited the sentence as below (also, see response # 4 above to the first reviewer):  **It is currently being adapted for adolescents in Botswana but has not been specifically adapted for ALWH.** |
| 3. Page 4-5: Study settings and participants & sample size   Comment: What is the justification for selecting 4 clinics? Please also a clarify if by patient volume you men these are high volume clinics? Are these public health clinics?  Please expand briefly on the Beck’s Depression Inventory: what is it and what does a score of ≥ 13 mean. Please add a justification for including those who scored ≥ 13 and why these are an important sub-group to include? Will the BDI-II be completed before enrolment or is this coming from a different data source- if this was previously completed, what was the cut-off date i.e. The score is valid for how long after the test was taken?  Please clarify eligibility criteria #4 “living in the clinic’s catchment area with intention to remain for at least 1 year” – what does “intention to remain” mean here? Does it refer to their place of residence within the clinic’s catchment area or remain receiving services from the clinic they were enrolled in?  What is the expected enrolment distribution per clinic and arm? How will you ensure that the enrolments are not skewed towards 1 clinic and that the study arms are not skewed by clinic enrolments? Are there any other socio-economic or structural differences across the 4 clinics that will need to be considered and how they could potentially influence the pilot results? | We thank the reviewer for raising these important points. We clarify these points below and add them to the text as appropriate.  Justification for selecting 4 clinics:  **Each of the four sites provide ART services and have a monthly ART clinic volume of more than 115 individuals (age 10-19) who are either initiating care, re-initiating care, or are established patients. We believe these clinic volumes will provide adequate numbers for our recruitment efforts. UNC Project has previously successfully recruited and enrolled adolescents in multiple longitudinal studies at these sites.**  BDI-II:  **The BDI-II consists of 21 items with a two-week recall period. Study staff will administer the BDI-II during the screening process. BDI-II scores are classified as minimal (0-13), mild (14-19), moderate (20-28), and severe (29-63). In a validation study amongst ALWH in Malawi, a BDI-II score of ≥13 achieved sensitivity of more than 80% [36].**  Intention to remain:  **(4) living in the clinic’s catchment area with intention to remain in their current residence place for at least 1 year (duration of study enrollment).**  Distribution per clinic and arm:  **We expect to enroll 105 total participants for this randomized pilot study (n=35 ALWH for each arm) with approximately 27 participants at each of the four study sites. Given participants will be randomized, we expect balance across arms, regardless of clinic.** |
| 4. Page 5: line 1 & 7 Comment: Please correct the abbreviation, it is written as “AWLH” instead of ALWH – please also check the whole manuscript, there are a few more similar errors. | We thank the reviewer for the thorough review. We double checked and edited this. |
| 5. Page 5: Intervention description Line 10 – Please add that AFB will be “individual” counselling sessions Line 10-11 – remove reference to “HIV clinics” – revise to refer to these as study clinics – in essence these are still clinics providing general health services with integrated ART? Line 37 – The frequency of the support session: please add a justification for holding monthly peer support sessions if the individual sessions are every 7-10 days. | We thank the reviewer for the thoughtful comment. We added “individual” and removed “HIV” in the sentence:  **The AFB will include 6 individual counseling sessions facilitated by counselors attached to one of the study clinics.**  Regarding the frequency of the sessions, we added this sentence in the paragraph describing AFB:  **We will conduct one counseling session every 7-10 days, instead of one session per week as in the original FB.**  For frequency of peer support, we revised:  **As suggested from aforementioned formative work, one group session per month is ideal for peer support component.** |
| General comments: in the delivery of the peer support intervention, are the younger adolescents (13-14/15 separated from the older adolescents (16-19) also separated by gender? How do you ensure inclusivity and maximum participation in the groups, the topics may be the same but engagement for younger and older adolescents and males may differ, being combined in the same session may lead to minimal participation from other age groups and gender? | We thank the reviewer for this comment. Peer support sessions will be conducted separately for younger (13-15) and older (16-19) ALWH. We decided not to separate the groups by gender given the preferences of ALWH in our formative research. |

**Reviewer 3**

| **Comment** | **Response** |
| --- | --- |
| General comments: Most importantly, there are no sample size calculations in this manuscript which must be included per PLOS ONE guidelines https://journals.plos.org/plosone/s/submission-guidelines#loc-study-protocols. This applies both on p.5 to the enrollment numbers but also to the subset that receive the qualitative evaluation.  In addition, I didn't realize there was a qualitative outcome until the data analysis section. There needs to be more detail on how these analyses will be performed. Listing out the steps gives a general idea, but I don't know how will coding be performed, or what sort of methods will be used.  I also could not locate a data management plan or a section on safety considerations in the manuscript.  Regarding the study design and analysis methods, one comment I have is, since the intervention was delivered in small groups, I believe this is an individual randomized group treatment trial (IRGT doi: 10.2105/AJPH.2007.127027). In IRGTs, the composition of the groups could influence the participant's outcome; in other words, within group responses may be correlated. If this is true, then group membership must be taken into account meaning the power will be lower than in an individually-randomized trial. | We thank the reviewer for the review and comments.  Regarding sample size calculation for our qualitative outcome, please see our below response (#3).  .  We added one sentence regarding data safety in the Ethnics and Confidentiality section.  Please refer to specific comments for clarification. |
| 1. (p.4, lines 21-22) Which software will you use? The random number generators are not all created equal. | We thank the reviewer for this comment. The randomization list is generated using the rand() function in Excel. |
| 2. (p.4, lines 23-24) I can see that blinding might not be possible, but I think this is too vague for a protocol. | We thank the reviewer for this comment. As the nature of counseling intervention, participants might or might not be aware of which type of intervention that they are receiving. Given that, we are trying to enroll participants with consistent contents of informed consent regardless of their assigned arm. Conservatively speaking, we suppose blinding might not be possible. |
| 3. (p.5, lines 1-3) No sample size calculations? See general comments. | We thank the reviewer for this comment. The main goal of this pilot RCT is to evaluate the feasibility, fidelity and acceptability of the interventions. Our secondary outcome is preliminary effectiveness. We are confident that the current sample size is enough to achieve this main goal and sample size calculation will be applied in a larger-scale RCT in the future. |
| 4. (p.6, line 17) How will you be sampling people for the exit interviews? Also, if these interviews are brief, why are they not performed with all people? | We thank the reviewer for raising this question. We will select conveniently a subset of enrolled participants for exit interviews and all providers (incl. counselors, peer supporter, clinical staff). We select participants based on their socio-demographic characteristics to make sure of the diversity of the qualitative sample. We aim to include 10 per arm (i.e., 30 in total). Given the rule of thumb of 5-10 in-depth interviews per stratum to achieve 80% of themes, we expect to achieve saturation within our proposed sample sizes.  We added this information in text (Page 7, in the paragraph of Acceptability outcome) and remove one sentence about the number of participants in qualitative part in Data collection section as we think it is more appropriate to move this part into paragraph explaining the outcome. |
| 5. (p.7, line 6) At no point before this is it mentioned that acceptability will be assessed qualitatively. This should be made clear in the methods section. I would also encourage you to split the outcomes into quantitative and qualitative as well and to be more clear about the qualitative methods that are to be used in this study. I also wonder whether 10 participants in each arm is sufficient for qualitative analysis. | We thank the reviewer for this comment. We first mention the qualitative piece in the “Primary and secondary outcomes” section. As we only use qualitative part as a small piece supported for one of the four outcomes in our study (i.e., acceptability), we seem to prefer keeping it embedded in the acceptability outcome description. |
| 6. (p.7) How will data be collected? Paper and pencil? ODK? A web-based system? How will data recording errors be minimized? | We thank the reviewer for the comment. Quantitative data will be collected and managed using Research Electronic Data Capture (REDCap). We added this information in the data collection section. |
| 7. (p.7, lines 18-21) I strongly encourage the authors to use standardized mean differences instead of p-values to compare baseline characteristics, especially in a small trial like this. Significance testing in these situations is generally frowned upon because a non-significant p-value does not indicate that groups are the same. For info on the topic in relation to baseline imbalance in randomized trials see Altman, https://doi.org/10.2307/2987510 and Senn, https://doi.org/10.1002/sim.4780131703. My recommendation is to use standardized difference to assess differences (see Austin, https://doi.org/10.1080/03610910902859574). | We thank the reviewer for this comment. As clarified earlier, the current pilot aims to characterize preliminary descriptions of the interventions. Comparing absolute count/numbers, proportions or continuous values might provide some sense of which parts could be of interest in adhoc analyses or future studies. That said, statistical tests or inferential considerations are less likely to be priorities of this pilot. Also, we are planning to use p values as it is probably intuitive for audiences. |
