# Supplementary material for: Friendship Bench intervention to address depression and improve HIV care engagement among adolescents living with HIV in Malawi: Study protocol for a pilot randomized controlled trial
Source: PLoS One. 2025 Mar 19;20(3):e0302666. doi: 10.1371/journal.pone.0302666 (PMC11922207; doi:10.1371/journal.pone.0302666)
Supplement: Attachment — Submitted filename: Response to Reviewers_21Nov2024_bng_tosubmit.docx [file pone.0302666.s005.docx]

November 21, 2024

Dear Reviewers,

We are pleased to resubmit our manuscript, “Friendship Bench Intervention to Address Depression and Improve HIV Care Engagement Among Adolescents Living with HIV in Malawi: Study Protocol for a Pilot Randomized Controlled Trial”. We greatly appreciate your thoughtful reviews and addressing your comments has strengthened our manuscript. We have responded to the comment about providing more details on sample size calculations and made changes to the manuscript of the tracked changes. Our detailed responses to the comment can be found on the table below.

Sincerely,

Authors

**Reviewer 1**

| **Comment** | **Response** |
| --- | --- |
| 1. Thank you, I am satisfied that the authors have incorporated my comments. Given that I have already reviewed the article, I have no further comments. | We thank the reviewer for the support. |

**Reviewer 2**

| **Comment** | **Response** |
| --- | --- |
| 1. The authors have made all the revisions suggested in the initial review process and provided sound justification where specific comments were not addressed. The revisions have strengthened the manuscript and it is clear how the protocol can be replicated. The study rationale is clear and provides evidence-based justifications. The study methods are described in detail and justifications provided for the site selection, sample selection and how the intervention will be delivered. There is no sample size calculation and the study is not powered, which may not be an issue since this is a pilot study and they do mention that these will be considered in a large-scale trial that will follow the this pilot. This is a protocol paper and no data is presented. Overall the manuscript is well written and the ideas flow in a logical manner. | We thank the reviewer for the support. |

**Reviewer 3**

| **Comment** | **Response** |
| --- | --- |
| 1. Regardless of the protocol aim or your confidence in your projected sample size, the PLOS ONE publication guidelines state that sample size calculations must be included. | We thank the reviewer for the comment. We made a subsection under the “Participants and sample size” section for “Sample size considerations”.  In the “Sample size considerations”, we added a justification for our sample size. The paragraph can be found in our revised manuscript and as below.  “As we explore feasibility, acceptability and fidelity of the interventions from a pilot study lenses, we do not calculate sample size for our main outcomes. The sample of 105 ALWH (35 per arm) will be sufficient to estimate quantitative measures of feasibility and acceptability with reasonable precision (e.g., confidence intervals around proportions of ±7-9 percentage points across all arms, and ±9-17 percentage points within a given arm).” |
| 2. Excel is not the best choice for random number generation, but it is passable so long as you use Excel 2010 or later. Previous versions used an algorithm with a short cycle length that had poor performance. Microsoft switched to an algorithm with a greater cycle length in 2010. Further details can be found here: https://link.springer.com/article/10.1007/s00180-014-0482-5. Unfortunately, you cannot set the seed in Excel, which means the numbers are not reproducible, but that should be ok for your purposes. | We thank the reviewer for your insights into randomization process. We specified the version of Excel in the manuscript and as below.  “The study team will generate a random sequence using computer-based software (the rand() function in Microsoft 365 Excel version 2410).” |
| 3. The problems with p-values are well documented (e.g., https://doi.org/10.1080/00031305.2016.1154108; http://www.stat.columbia.edu/~gelman/research/published/asa_pvalues.pdf;https://doi.org/10.1080/02664763.2011.567245; https://doi.org/10.1098/rsos.171085; https://doi.org/10.1007/s10654-016-0149-3) and I would argue that they are decidedly NOT intuitive since so many misinterpretations exist. If inferential considerations are not a priority of the manuscript, then why invite inferential conclusions to be made by including p-values? | We thank the reviewer for your comment. We highly appreciate the suggestion to strengthen our statistical analysis. We will take standardized mean differences into account to compare continuous variables across study arms in the data analysis phase of this study. |
